# Astodrimer sodium and bacterial vaginosis: a mini review

**DOI:** 10.1007/s00404-022-06429-z

**Published:** 2022-03-04

**Authors:** Werner Mendling, Wolfgang Holzgreve

**Affiliations:** 1 German Centre for Infections in Gynecology and Obstetrics at Landesfrauenklinik, Helios University Hospital Wuppertal, Heusnerstrasse 40, 42283 Wuppertal, Germany; 2 Wolfgang Holzgreve, University Hospital Bonn, Venusberg-Campus 1, 53127 Bonn, Germany

**Keywords:** Bacterial vaginosis, Astodrimer sodium, Vaginal gel, Non-antibiotic treatment

## Abstract

Bacterial vaginosis (BV) is the most common vaginal infection affecting women of childbearing age, and is associated with a substantial burden on women’s physical, emotional, sexual and social lives, as well as being linked to a number of gynaecological and obstetrical complications and adverse pregnancy outcomes. Antibiotics, such as metronidazole or clindamycin, are recommended as first-line treatment for BV, but may be associated with antibiotic resistance, high rates of recurrence and poor patient treatment satisfaction. Astodrimer sodium gel is a novel, non-antibiotic treatment for BV that is not systemically absorbed. It prevents pathogenic bacteria from adhering to the vaginal wall, and disrupts and inhibits the formation of pathogenic bacterial biofilms. Clinical cure rates of 50–57% were observed in patients with BV treated with astodrimer sodium compared with 17–21% treated with placebo (*p* < 0.001) in Phase 3 trials. In a separate Phase 3 trial, recurrence of BV occurred in 44% of patients treated with astodrimer sodium compared with 54% of patients who received placebo (*p* = 0.015). Astodrimer sodium is well tolerated, with vulvovaginal candidosis being the only treatment-related adverse event reported to occur more often than with placebo. The availability of astodrimer sodium, a well-tolerated, convenient, non-antibiotic treatment for BV, represents significant progress in the treatment of this burdensome condition.

## Introduction

Bacterial vaginosis (BV) is the most common vaginal syndrome affecting women of childbearing age [1, 2]. It is characterised by foul-smelling vaginal discharge and may sometimes also be accompanied by dysuria, dyspareunia, burning and vaginal inflammation [3, 4]. Additionally, BV is a major cause of complications affecting the reproductive health of women [1, 5, 6].

Oral or intravaginal antibiotics are currently recommended as first-line treatment for BV, including persistent or recurrent BV [7, 8]. However effective, there is potential for development of antibiotic resistance with these treatments [9–11]. In addition, BV symptoms often recur soon after treatment has ended, with patients reporting low treatment satisfaction and a dislike of taking antibiotics [12]. Non-antibiotic treatments and probiotics have been investigated for the treatment of BV with varying degrees of success [13–15].

More recently, a number of clinical trials investigating a novel non-antibiotic treatment, astodrimer sodium, have been published, and astodrimer sodium is now available for the treatment and prevention of recurrent BV [16–18]. This mini review will provide a brief overview of the epidemiology and pathophysiology of BV before summarising the findings of the astodrimer sodium clinical trials.

## Pathophysiology and epidemiology of bacterial vaginosis

### Pathophysiology of bacterial vaginosis

The ‘normal’ vaginal flora comprises a mixture of more than five hundred species of bacteria kept in balance by different lactobacilli [19, 20]. These bacteria provide numerical dominance, preventing harmful bacteria from gaining a foothold [1]. The most prevalent are usually *Lactobacillus* species [20–23], but the distribution of species varies significantly between ethnicities and between women of the same ethnicity [24–26]. Lactobacilli produce lactic acid and hydrogen peroxide that maintain a normal, acidic vaginal pH (3.5–4.5); the acidic environment inhibits the growth of pathogens and protects against infections [20, 27].

A wide range of factors, including sexual habits, smoking and personal hygiene habits [20, 28], can increase the vaginal pH, making conditions unfavourable for Lactobacilli and allowing the growth of predominantly anaerobic bacteria such as *Gardnerella vaginalis*, resulting in BV [27]. Some *G. vaginalis* strains can then form a biofilm with other bacteria [29, 30], which provides protection from lactic acid and hydrogen peroxide [31], and can lead to recurrent episodes of BV.

### Signs and symptoms of bacterial vaginosis

It is estimated that 50–75% of women with BV are asymptomatic [32–35]; those with symptoms typically present with an off-white, thin, homogeneous vaginal discharge and a “fishy” vaginal odour [35, 36]. Patients may also have vulvar or vaginal pruritus, burning and irritation [3, 36].

### Diagnosis of bacterial vaginosis

Diagnosis of BV is generally made using the Amsel clinical criteria [32] or the laboratory-based Nugent scoring system [37].

The Amsel clinical criteria require three of the following four symptoms or signs for a positive diagnosis: an adherent, homogeneous, white discharge that coats the vaginal wall; a raised vaginal pH of > 4.5; a positive “whiff amine test” upon addition of potassium hydroxide to the discharge and/or the presence of clue cells upon the wet mount [32]. Clue cells are vaginal epithelial cells covered by adherent gram-negative rods [38].

Nugent’s scoring system assesses the number of *Gardnerella* morphotypes, gram-variable rods, and *Lactobacillus* morphotypes, giving an overall score out of 10 that predicts the likelihood of BV [37]. A score of 7 or more is indicative of BV.

### Epidemiology of bacterial vaginosis

According to a recent meta-analysis, BV is estimated to affect 23–29% of reproductive-aged women in the general population worldwide [2]. Globally, there are considerable differences between geographic regions and ethnic populations, with the highest prevalence in South Asian women (28.7%) and the lowest prevalence in European and Central Asian women (22.8%). Within North America, black and Hispanic women had significantly higher BV prevalence (33.2% and 30.7%, respectively) than other ethnic groups (white: 22.7%; Asian: 11.1%; *p* = 0.001). The prevalence of BV among pregnant women was similar to the general population overall (11.7–33.2%), while it was higher among women living with human immunodeficiency virus (35.6% vs 25.6%; *p* = 0.054) [2].

Risk factors for BV include new and higher numbers of male and female partners, young age of first intercourse [39–44], lack of condom use [39, 45], presence of other sexually transmitted infections [44], smoking [28, 43], drinking alcohol [28], and frequent douching/vaginal cleansing [28, 42, 43, 45].

### Burden and complications of bacterial vaginosis

Around two-thirds of women with recurrent BV report a moderate to severe impact on their physical, emotional, sexual and social lives [3]. Malodour is reported to be the most distressing symptom, although many women also find the discharge distressing. Women with recurrent BV reported feeling embarrassed, self-conscious and uncomfortable, and they reported that they were always worrying about BV. The biggest impact of BV was on women’s sex lives and practices, with sexual self-esteem, confidence and levels of intimacy also affected [3].

Recurrent BV is common, with 58–76% of women who have undergone metronidazole treatment reporting recurrence within 12 months [46–48]. A study aiming to predict the likelihood of recurrence showed that a higher pre-treatment abundance of *Lactobacillus* spp. relative to BV-associated species was linked with a higher likelihood of recurrence due to sequestration of metronidazole [49]. Conversely, another study found that persistently high titres of *Gardnerella Gsp07* and/or *G. swidsinskii* / *G. leopoldii* were associated with refractory/recurrent BV [50].

As well as being associated with poor levels of effectiveness, women reported frustration and dissatisfaction with current available treatments for BV [12]. In addition, women reported low levels of satisfaction with the clinical management of BV, including inconsistent advice, misdiagnosis, inappropriate diagnostic approaches and insensitive or dismissive attitudes. These frustrations led many women to try self-help remedies and lifestyle modifications, including the high-risk practice of douching.

BV is associated with a number of obstetric complications and adverse pregnancy outcomes. In various studies, BV has been associated with an increased risk of preterm delivery [51–55], premature rupture of membranes [55], low birthweight [52, 55], early spontaneous abortion [51, 56, 57], late miscarriage [53], and maternal infection [51, 53, 55]. In addition, BV has been associated with pelvic inflammatory disease, including endometriosis [6, 58], infertility [6, 56, 59], and sexually transmitted diseases [5, 58, 60–62].

## Guidelines for the treatment of bacterial vaginosis

Guidelines from the International Union against Sexually Transmitted Infection (IUSTI) and World Health Organization (WHO) on the management of vaginal discharge were published in 2018 and address BV [7]. The guidelines recommend 5–7 days of oral metronidazole 400–500 mg twice daily, intravaginal metronidazole gel (0.75%) once daily for 5 days, or intravaginal clindamycin cream (2%) once daily for 7 days, as first-line therapy for uncomplicated BV (grade 1 recommendation; grade A quality of evidence) [7]. Alternative regimens include metronidazole 2 g orally in a single dose, tinidazole 2 g orally in a single dose or 1 g orally for 5 days, clindamycin 300 mg orally twice daily for 7 days, or dequalinium chloride 10 mg vaginal tablets once daily for 6 days. For recurrent and persistent BV, IUSTI/WHO guidelines recommend that the current best treatment is intravaginal metronidazole, but the strength of recommendation is grade 2, and the quality of evidence is grade B [7].

Other relevant guidelines include those of the Association of the Scientific Medical Societies in Germany (*AWMF;* Arbeitsgemeinschaft der Wissenschaftlichen Medizinischen Fachgesellschaften), which were published in 2014. These guidelines also recommend oral or vaginal metronidazole or intravaginal clindamycin cream [8]. They also report evidence for prevention of recurrence with dequalinium chloride vaginal tablets and nifuratel vaginal tablets [8].

## Astodrimer sodium vaginal gel for bacterial vaginosis

### Mechanism of action

Astodrimer sodium is a dendrimer, a class of compounds characterised by a highly branched, three-dimensional architecture [16]. The core of astodrimer sodium is made of the benzhydrylamine amide of L-lysine, to which four successive layers of L-lysine branching units are added, creating a dendrimer with 32 amine groups on the surface. Finally, sodium 1-(carboxymethoxy) naphthalene-3,6-disulphonate groups are attached to each of the amine surface groups [63, 64]. This process results in a large molecule (16,581 Da) with a negative surface charge, which is not systemically absorbed [16, 63].

**Fig. 1:**
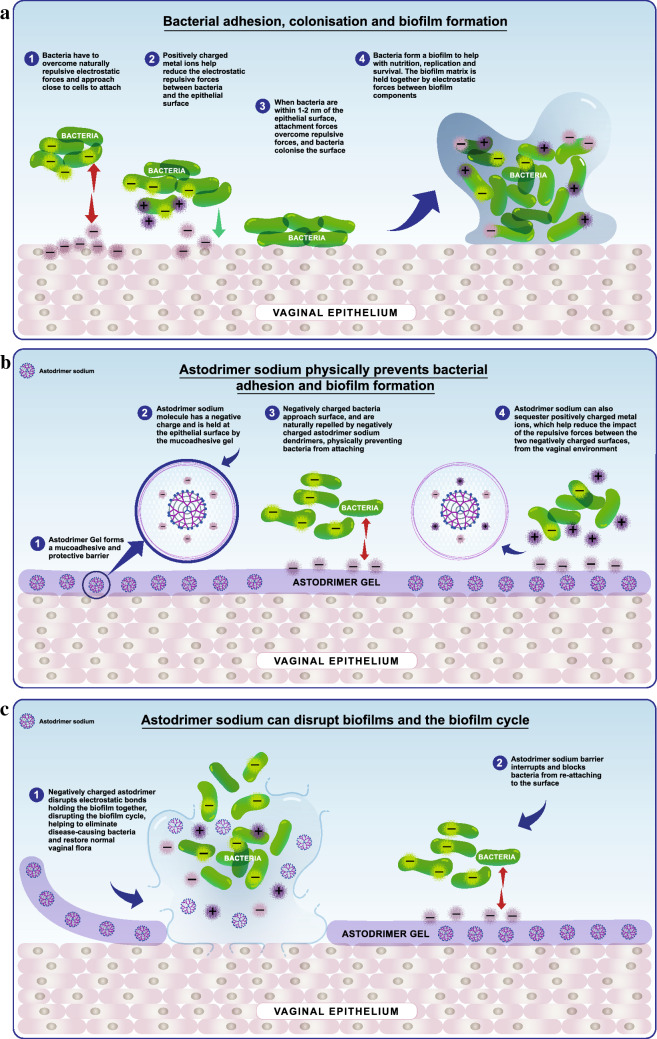
Antibacterial mechanism of action of astodrimer sodium. **a** Bacteria attach to and colonise the vaginal epithelium, forming a biofilm. **b** Astodrimer Gel containing astodrimer sodium, forms a barrier that blocks bacteria from attaching to the vaginal epithelium. **c** Astodrimer sodium also disrupts biofilms. © Starpharma Pty Ltd, 2021

Astodrimer sodium is formulated in an aqueous, Carbopol^®^-based, muco-adhesive gel [63]. It inhibits the growth of bacteria associated with BV by blocking their attachment to cells, and can inhibit the formation of, and disrupt existing, biofilms (Fig. 1). As noted earlier, biofilms are an important factor in the pathogenesis of BV, and are not well managed by existing therapies. This situation leads to inadequate treatment and the potential for recurrence [16]. Preclinical studies in a range of in vitro and animal models demonstrated that astodrimer was non-toxic at clinically relevant doses and well tolerated [65–68].

### Astodrimer sodium for the treatment of bacterial vaginosis

Astodrimer sodium was investigated as a treatment for BV in a Phase 2 study that enrolled 132 women with BV who were randomised to astodrimer sodium 0.5%, 1%, or 3%, or hydroxyethyl cellulose placebo gel at a dose of 5 g vaginally once daily for 7 days. The primary endpoint was clinical cure at study Days 21–30 [16].

Clinical cure rates at Day 21–30 were 28.0%, 46.2%, 23.3%, and 11.5% with the 0.5%, 1%, and 3% astodrimer doses, and placebo, respectively (*p* = 0.006 for 1% gel vs placebo). At Day 9–12, clinical cure rates with astodrimer sodium were superior to placebo, with rates of 62.5%, 74.1%, 55.2%, and 22.2% with the 0.5%, 1%, and 3% astodrimer doses, and placebo, respectively (*p* < 0.001 for 1% gel vs placebo) [16].

Adverse events (AEs) considered possibly related to treatment occurred in 25.0%, 18.8%, and 31.0% of astodrimer 0.5%, 1%, and 3% gel-treated patients, respectively, and 21.9% of placebo patients. Patients found astodrimer gel to be acceptable and were satisfied with treatment, as shown by significantly higher scores compared with placebo on the Treatment Satisfaction Questionnaire for Medicine (TQSM) for effectiveness and Global Satisfaction. Scores for convenience and tolerability were similar between the astodrimer and placebo groups [16].

Two Phase 3 studies were conducted to confirm the efficacy and safety of astodrimer sodium 1% gel for the treatment of BV. Study 1 was conducted in the US and Study 2 was conducted in the US, Germany and Belgium [17]. In both studies, patients were randomised 1:1 to astodrimer 1% gel or placebo at a dose of 5 g vaginally once daily for 7 days. In Study 1, 127 patients were randomised to astodrimer and 123 to placebo, and in Study 2, 128 patients were randomised to astodrimer and 123 to placebo. The primary endpoint was clinical cure at Day 9–12.

Astodrimer was superior to placebo for the primary endpoint and some of the secondary endpoints. Clinical cure rates at Day 9–12 were 50.4% vs 16.5% (*p* < 0.001; Study 1) and 56.7% vs 21.4% (*p* < 0.001; Study 2) for astodrimer compared with placebo. Nugent cure rates at Day 9–12 were 12.8% vs 2.6% (*p* = 0.004; Study 1) and 13.3% vs 5.1% (*p* = 0.030; Study 2) for astodrimer compared with placebo. The differences between the astodrimer and placebo groups were smaller at the follow-up visit on Day 21–30, but still favoured astodrimer and were statistically significant in some cases. More women receiving astodrimer reported absence of vaginal discharge and absence of vaginal odour at Day 21–30 compared with placebo (discharge: 52.1% vs 37.4%, *p* = 0.023 for Study 1, 60.0% vs 50.4%, *p* = 0.131 for Study 2; odour: 52.1% vs 44.3%, *p* = 0.222 for Study 1, 63.3% vs 37.4%, *p* = 0.006 for Study 2). When the primary analysis of clinical cure at Day 9–12 was conducted in patients with a baseline Nugent score ≥ 7, astodrimer was also superior to placebo: 52.7% vs 17.3%, *p* < 0.001 in Study 1; and 57.5% vs 14%, *p* < 0.001 in Study 2 [17].

The studies were combined to assess safety and tolerability. The overall incidence of AEs was 42.9% for astodrimer and 41.4% for placebo, and AEs possibly related to treatment were reported by 14.7% of patients who received astodrimer and 9.4% of patients who received placebo. The most common AEs reported, irrespective of relationship to treatment, were headache (7.5% in the astodrimer group and 7.0% in the placebo group), vulvovaginal candidosis (6.0% and 3.7%, respectively) and vulvovaginal pruritus (5.2% and 4.5%, respectively). Vulvovaginal candidosis considered potentially related to study treatment occurred in 2.4% of astodrimer-treated patients but no placebo-treated patients. Urinary tract infections (irrespective of treatment relationship) were reported in 1.6% of the astodrimer group and 1.2% of the placebo group [17].

Following the publication of these data, a meta-analysis was conducted to examine the efficacy and safety of astodrimer gel for BV [69]. For efficacy outcomes, it included the Phase 2 and 3 studies discussed above. The meta-analysis found that astodrimer gel was significantly superior to placebo for all pooled efficacy outcomes, including: clinical cure rate [pooled risk ratio (RR) 2.10, 95% confidence interval [CI] 1.76–2.51; *p* < 0.01]; microbiological Nugent cure rate (RR 4.41, 95% CI 2.49–7.81; *p* < 0.01); patient self-reported absence of vaginal odour (RR 1.57 95% CI 1.40–1.77; *p* < 0.01) and discharge (RR 1.45, 95% CI 1.29–1.64; *p* < 0.01); resolution of Amsel criteria (all criteria *p* < 0.01) and proportion of patients who did not require rescue therapy (RR 1.68, 95% CI 1.13–2.51; *p* = 0.01).

The safety outcomes were investigated in the three treatment studies and the prevention of recurrence Phase 3 study discussed below [18]. The meta-analysis found that astodrimer had similar tolerability to placebo for all pooled safety endpoints with the exception of vulvovaginal candidosis (RR 1.427, 95% CI 1.025–1.986; *p* = 0.035) and treatment-related vulvovaginal candidosis (RR 1.181, 95% CI 1.020–3.239; *p* = 0.043). When compared with placebo, the incidence of severe AEs was significantly lower in the astodrimer group (RR 0.373, 95% CI 0.146–0.950; *p* = 0.039) [69].

### Astodrimer sodium for prevention of recurrent bacterial vaginosis

In order to demonstrate the efficacy and safety of astodrimer sodium 1% gel to prevent recurrence of BV, a large Phase 3 study was conducted [18]. A total of 864 women with BV and a history of recurrent BV were enrolled and received oral metronidazole 500 mg twice daily for 7 days. Successfully-treated women were then randomised 1:1 to receive astodrimer sodium 1% gel or placebo at a dose of 5 g vaginally every second day for 16 weeks, followed by a further 12 weeks off-treatment. The primary endpoint was recurrence of BV (presence of ≥ 3 Amsel criteria) at or by Week 16 [18].

Astodrimer sodium was superior to placebo for the primary and many of the secondary endpoints. For the primary endpoint, recurrence occurred in 44.2% of patients who received astodrimer compared with 54.3% of patients who received placebo (*p* = 0.015). Time to recurrence of BV was significantly longer for women receiving astodrimer compared with placebo, with Kaplan–Meier curves separating after Week 4 and remaining so until Week 16 (*p* = 0.007). Recurrence of all individual Amsel criteria at or by Week 16 was lower in the astodrimer group compared with placebo with the exception of vaginal fluid pH. Recurrence of subject-reported symptoms at or by Week 16 was also significantly lower in the astodrimer group versus placebo (vaginal discharge in 20.4% vs 28.6%, RR 0.71, 95% CI 0.53–0.96, *p* = 0.025; vaginal odour in 20.7% vs 31.5%, RR 0.66, 95% CI 0.49–0.88, *p* = 0.004) [18].

During the 12-week follow-up phase, recurrence of BV was lower in the astodrimer group than the placebo group, but the differences were not statistically significant. Recurrence of BV symptoms of vaginal odour and/or discharge was significantly lower in the astodrimer arm versus placebo up to 8 weeks after cessation of therapy (Week 24, 36.1% vs 45.5%, *p* = 0.027) [18].

The overall incidence of AEs was 54.1% with astodrimer and 47.4% with placebo. Potentially treatment-related AEs occurred in 12.6% of astodrimer-treated patients and 11.3% of placebo-treated patients. The most common AEs during treatment were vulvovaginal candidosis (18.0% with astodrimer and 13.7% with placebo), urinary tract infection (7.8% and 2.4%) and headache (5.1% and 6.2%). Potentially treatment-related vulvovaginal candidosis was reported in 6.8% and 4.8% of astodrimer- and placebo-treated patients, respectively. During follow-up, vulvovaginal candidosis rates were 4.1% and 5.8% for astodrimer- and placebo-treated patients, respectively [18].

## Conclusions

This mini-review has highlighted an unmet need for new treatment options in BV, the most common vaginal syndrome affecting women of childbearing age [1, 2]. While first-line treatment with antibiotics is recommended and supported by robust evidence [7, 8], there remains potential for the development of antibiotic resistance with repeated exposure to these medications [9] and resistance to polybacterial biofilms in both/all sexual partners [29]. In addition, some patients have reported poor treatment satisfaction and dislike taking antibiotics, particularly on a regular basis [12].

Astodrimer sodium gel is a non-antibiotic treatment for BV that acts locally via a novel mechanism of action by which it inhibits the formation of, and disrupts existing, biofilms [16]. It is not systemically absorbed and is formulated in a muco-adhesive gel [16]. It treats BV, restores vaginal flora balance, and normalises vaginal pH; it also effectively prevents recurrent BV and its symptoms [70]. Astodrimer sodium provides rapid relief of vaginal discomfort such as unpleasant odour and discharge, and offers a convenient once daily or every second day vaginal application, depending on the indication [70].

The use of Astodrimer sodium gel in the treatment and prevention of BV is supported by high-quality clinical trial data demonstrating superior efficacy compared with placebo [16–18]. It is also well tolerated in terms of AEs, and was acceptable to women in terms of treatment satisfaction [16–18].

### Future perspectives

Astodrimer sodium has the potential to improve outcomes for patients with BV, as it is a non-antibiotic treatment with no potential to cause antibiotic resistance, is not systemically absorbed and is also convenient. It is formulated as a transparent gel that adheres to the vaginal wall, thus avoiding leakage from the vagina, which can be an inconvenience associated with other topical treatments. The availability of a well-tolerated, convenient non-antibiotic treatment for BV represents significant progress in the treatment of BV and may benefit women affected by this widespread condition.

## Data Availability

All referenced publications are in the public domain.
